# Functional Connectome of the Five-Factor Model of Personality

**DOI:** 10.1017/pen.2017.2

**Published:** 2018-05-25

**Authors:** Nicola Toschi, Roberta Riccelli, Iole Indovina, Antonio Terracciano, Luca Passamonti

**Affiliations:** 1 Department of Biomedicine & Prevention, University “Tor Vergata”, Rome, Italy; 2 Department of Radiology, Martinos Center for Biomedical Imaging, Boston & Harvard Medical School, Boston, MA, USA; 3 Laboratory of Neuromotor Physiology, IRCCS Santa Lucia Foundation, Rome, Italy; 4 The Centre of Space BioMedicine, University of Rome Tor Vergata, Rome, Italy; 5 Department of Geriatrics, Florida State University College of Medicine, Tallahassee, FL, USA; 6 Institute of Bioimaging & Molecular Physiology, National Research Council, Catanzaro, Italy; 7 Department of Clinical Neurosciences, University of Cambridge, Cambridge, UK

**Keywords:** Big Five, individual differences, resting-state FMRI, connectome, graph analysis

## Abstract

A key objective of the emerging field of personality neuroscience is to link the great variety of the enduring dispositions of human behaviour with reliable markers of brain function. This can be achieved by analysing big data-sets with methods that model whole-brain connectivity patterns. To meet these expectations, we exploited a large repository of personality and neuroimaging measures made publicly available via the Human Connectome Project. Using connectomic analyses based on graph theory, we computed global and local indices of functional connectivity (e.g., nodal strength, efficiency, clustering, betweenness centrality) and related these metrics to the five-factor model (FFM) personality traits (i.e., neuroticism, extraversion, openness, agreeableness, and conscientiousness). The maximal information coefficient was used to assess for linear and nonlinear statistical dependencies across the graph “nodes”, which were defined as distinct large-scale brain circuits identified via independent component analysis. Multivariate regression models and “train/test” approaches were used to examine the associations between FFM traits and connectomic indices as well as to assess the generalizability of the main findings, while accounting for age and sex variability. Conscientiousness was the sole FFM trait linked to measures of higher functional connectivity in the fronto-parietal and default mode networks. This offers a mechanistic explanation of the behavioural observation that conscientious people are reliable and efficient in goal-setting or planning. Our study provides new inputs to understanding the neurological basis of personality and contributes to the development of more realistic models of the brain dynamics that mediate personality differences.

Personality neuroscience is a rapidly growing research field that aims at understanding the neural underpinnings of variability in cognitive and emotional functions as well as the brain basis of individual differences in behaviour (Corr, 2006; DeYoung, Hirsh, Shane, Papademetris, Rajeevan, & Gray, 2010). Extensive research in personality has shown that the complexity of human behaviour can be described by an aggregate taxonomy termed the five-factor model (FFM) (Costa & McCrae, 1992; Digman, 1990; McCrae & Terracciano, 2005), although other models of personality have also been developed to explain a wide range of behaviours, including clinical disorders, occupational/educational performance, and economic choices (Ashton et al., 2004; Cloninger, 1999; Cloninger, Przybeck, & Svrakic, 1991; Cloninger, Svrakic, & Przybeck, 1993; Corr, 2006; Eysenck, 1983, 2012; Gray, 1970; Gray & McNaughton, 2003). The FFM posits that neuroticism, extraversion, openness, agreeableness, and conscientiousness are universal descriptors of the human enduring behavioural dispositions (McCrae, 1991; McCrae & Costa, 1987; McCrae & John, 1992; McCrae & Terracciano, 2005).

However, *how* individuals differ in these traits remain an important open question. Recently, sophisticated brain imaging techniques and new analytical methods have become available to formulate novel models regarding the neurological basis of human personality, although it must be acknowledged that neuroimaging is an indirect and correlational measure of brain anatomy and function. Past research has linked the FFM traits to different indices of brain structure and function, although the presence of mixed and often conflicting results in the literature limits the conclusions that can be drawn from these studies (Canli, 2004; Canli, Sivers, Whitfield, Gotlib, & Gabrieli, 2002; Cremers et al., 2010, 2011; DeYoung et al., 2010; Dima, Friston, Stephan, & Frangou, 2015; Fischer, Wik, & Fredrikson, 1997; Hu et al., 2011; Indovina, Riccelli, Staab, Lacquaniti, & Passamonti, 2014; Kapogiannis, Sutin, Davatzikos, Costa, & Resnick, 2012; Krebs, Schott, & Duzel, 2009; Liu et al., 2013; Lu et al., 2014; Passamonti et al., 2015; Riccelli, Indovina, et al., 2017; Rodrigo et al., 2016; Servaas et al., 2013; Wright, Feczko, Dickerson, & Williams, 2007; Wright et al., 2006). Several factors may explain the inconsistences across previous findings, including the use of different analytic approaches and the fact that most of the earlier studies, with some notable exceptions (Bjornebekk et al., 2013; Holmes et al., 2012; Nostro, Muller, Reid, & Eickhoff, 2016; Riccelli, Toschi, Nigro, Terracciano, & Passamonti, 2017), have been conducted in small samples of participants.

Another important issue is the necessity to progress from accounts that describe personality differences in terms of anatomical and functional heterogeneity in isolated brain regions, to formal frameworks that model the complexity of the connectivity patterns at the whole-brain circuit level. Within this context, mathematical approaches based on graph theory have been developed to measure the architecture (“topology”) of the brain structural and functional connectivity (i.e., “connectomic” approaches) (Fornito & Bullmore, 2015). The graph theoretical approach provides a series of indices that quantify different aspects of the brain “connectome” (Fornito & Bullmore, 2015). For instance, the network’s capacity to “route” information across its elements (“nodes”) can be estimated by computing the efficiency of the paths (“edges”) linking these nodes (Boccaletti, Latora, Moreno, Chavez, & Hwang, 2006). In other words, the network’s efficiency is a quantitative representation of “how easy” it is for an input to “travel” across the graph’s nodes. Consequently, increased efficiency reflects heightened capacity of a network to process and route relevant information across its nodes. Graph analyses also enable to quantify the degree of segregation of a network (modularity) and its capacity to integrate the information at a global or local level (i.e., global or local clustering coefficient) (Rubinov & Sporns, 2010).

Studying how “communications” across large-scale brain circuits relate to each of the FFM traits has thus the potential to improve our understanding of the neurological roots of human personality. The rationale behind this study was to associate each of the FFM traits with functional connectivity patterns across large-scale brain networks. Although the relationship between the blood-oxygen-level-dependant activity in single regions and the whole-brain network measures is highly complex, there is evidence that “holistic” neuroimaging approaches are able to predict individual variability in multiple behavioural, demographic, and lifestyle measures (Smith et al., 2015). However, it remains to be determined whether graph-based metrics can be associated to individual differences in the FFM personality traits. To take a step in this direction, we studied the brain functional connectome in relation to the FFM in a large sample of individuals drawn from the Human Connectome Project (HCP) (*n*=818, age range: 22–37 years). The HCP is an international project that has granted open access to an unprecedented large set of demographics, personality, and neuroimaging data with high spatial and temporal resolution (McNab et al., 2013).

By using robust and highly validated methods to analyse resting-state functional magnetic resonance imaging (rs-fMRI) data, we tested how individual differences in neuroticism, extraversion, openness, agreeableness, and conscientiousness were associated to global and local indices of brain functional connectivity (e.g., nodal strength, efficiency, clustering). A validation approach based on a “training” and “testing” split of the total data set was also employed to assess for the replicability of the main findings. We hypothesized that the FFM traits linked to less favourable outcomes (e.g., risk of developing psychiatric disorders) like neuroticism were associated to reduced brain functional connectivity (e.g., low nodal strength, low clustering, and low efficiency). Conversely, FFM traits like openness, extraversion, agreeableness, and conscientiousness (which have been linked to curiosity, social skills, and life success) were expected to relate to measures of heightened functional connectivity (e.g., high nodal strength, high clustering, and high efficiency).

These predictions were based upon a recent study which found that functional connectomic metrics relate to a “single-axis” covariation (ranging from “positive” to “negative” measures) in behavioural traits (Smith et al., 2015). In other words, those individuals scoring high on the “positive” end of the behavioural axis linking lifestyle, demographic, and other psychometric measures (e.g., fluid intelligence) displayed stronger functional connectivity patterns than low-scoring participants (Smith et al., 2015). Interestingly, the brain regions that most contributed to these increased functional connectivity patterns included those areas that belong to the default mode network (DMN) (e.g., the medial prefrontal cortex, posterior cingulate, and temporo-parietal junction). Although the precise role of each region within the DMN is still matter of debate (Leech, Kamourieh, Beckmann, & Sharp, 2011), there is robust evidence that the DMN *as a whole* is involved in several aspects of human cognition and behaviour, including episodic and semantic memory, imagination, decision-making, and theory of mind (Roberts et al., 2017; Schacter, 2012; Schacter et al., 2012; Schacter, Benoit, De Brigard, & Szpunar, 2015). It is thus reasonable to expect that enhanced functional connectivity patterns within and across the DMN is linked with FFM personality traits that predict “positive” and favourable behavioural outcomes, although caution is always warranted when making reverse inferences in interpreting neuroimaging findings (Poldrack, 2006).

## Participants and methods

### Participants

The demographic and personality variables of the HCP sample are summarized in Table 1.

**Table 1:** Demographic and personality variables in the Human Connectome Project sample (*n*=818 volunteers)

Demographic variables	
Gender (males/females)	367/451
Age (years)	28.7±3.7 [22–37]
Handedness (right/left/both)	743/73/2
Education (years)	14.9±1.8 [11–17]
Ethnicity (%)	
Hispanic/Latino	8.6%
Not Hispanic/Latino	90.5%
Unknown/Not Reported	0.9%
Personality scores (NEO-FFI)	
Neuroticism	16.3±7.2 [0–43]
Extraversion	30.7±5.9 [11–47]
Openness	28.3±6.1 [12–45]
Agreeableness	32.0±5.0 [13–45]
Conscientiousness	34.5±5.9 [12–48]

*Notes*: NEO-FFI=NEO five-factors inventory questionnaire. Age, education, and personality data are expressed as mean±standard deviation, whereas the range in parentheses is expressed as minimum–maximum.

### Personality assessment

The FFM personality traits were assessed via the NEO five-factor inventory (NEO-FFI) (Costa & McCrae, 1992; Terracciano, 2003). The NEO-FFI is composed by 60 items, 12 for each of the five factors. For each item, participants reported their level of agreement on a 5-points Likert scale, from strongly disagree to strongly agree. The NEO instruments have been previously validated in the United States and several other countries (McCrae & Terracciano, 2005).

### MRI scanning protocol and preprocessing

rs-fMRI data were acquired from a 3T scanner (Siemens AG, Erlangen, Germany) (Van Essen et al., 2012). Four runs of 15 min each were obtained. Participants lay within the scanner with open eyes while fixating a bright central cross-projected on a dark background. Oblique axial acquisitions were alternated between phase encoding in a right-to-left direction in one run and phase encoding in a left-to-right direction in the other run. Gradient-echo echo-planar imaging used the following parameters: repetition time (TR)=720 ms, echo time (TE)=33.1 ms, flip angle=52°, field of view (FOV)=208×180 mm, matrix 104×90, slice thickness=2.0 mm, 72 slices, 2.0 mm isotropic voxels, multiband factor=8, echo spacing=.58 ms, bandwidth (BW)=2,290 Hz/Px. There were 4,800 rs-fMRI volumes in total per participant, subdivided in four runs of 1,200 volumes each. Structural (T1-weighted) images and field maps were also acquired to aid data preprocessing.

Each 15-min (1,200 volumes) run of each participant’s rs-fMRI data were preprocessed using FMRIB Software Library (FSL; https://fsl.fmrib.ox.ac.uk/fsl/fslwiki/) and it was minimally preprocessed according to the latest version (3.1) of the HCP pipeline (Glasser et al., 2013). Each data set was then temporally demeaned and had variance normalization applied according to Beckmann and Smith (2004). Group-principal component analysis (PCA) output was generated by MIGP (MELODIC’s Incremental Group-PCA), a technique that approximates full temporal concatenation of all participants’ data, from all 818 participants. This comprises the top 4,500 weighted spatial eigenvectors from a group-averaged PCA (Smith, Hyvärinen, Varoquaux, Miller, & Beckmann, 2014). The MIGP output was then fed into group-independent components analysis (ICA) using FSL’s MELODIC tool (Beckmann & Smith, 2004), applying spatial-ICA at dimensionality of 15. Successively, the ICA maps were dual regressed into each participant’s four-dimensional data set to give a set of 15 time courses of 4,800 time points per participant. Further details regarding data acquisition and processing can be found in the HCP S900 Release reference manual available at https://www.humanconnectome.org/

### Estimation of functional connectivity

To quantify the resting-state functional connectivity among the 15 circuits (“nodes”), the maximum information coefficient (MIC) between the time series of each pair of circuits was computed (Reshef et al., 2011). MIC is a powerful statistical measure that is sensitive to both linear and nonlinear associations of arbitrary shape between paired variables (Reshef et al., 2011). This method has been applied to investigate the functional connectivity patterns in patients with schizophrenia (Su, Wang, Shen, Feng, & Hu, 2013; Zhang, Sun, Yi, Wu, & Ding, 2015). The basic idea underlying MIC is that, when a relationship between two variables exists, it can be quantified via creating a grid on the scatterplot that creates a partition of the data. More formally, the MIC between two variables *x* and *y* is defined as
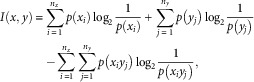
where *n*_*x*_ and *n*_*y*_ are the number of bins of the partition of the *x*- and *y*-axis. Therefore, the MIC of two variables *x* and *y* is calculated as
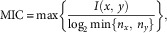
where the maximum is taken over all the possible *n*_*x*_ by *n*_*y*_ grids. The MIC between each pair of networks’ time series was calculated using the MINEPY toolbox (Albanese et al., 2013) implemented in MATLAB (https://github.com/minepy/minepy). These analytical steps generated a 15×15 full and symmetric subject-specific matrix of functional connectivity data. The matrices were then treated as weighted networks to calculate the graph-related measures.

### Local network analyses

All graph measures were computed via the Brain Connectivity Toolbox (Rubinov & Sporns, 2010) in MATLAB (https://sites.google.com/site/bctnet/). For each ICA and at the participant level, we calculated the graph measures that quantify the centrality of a node within a network (local strength and betweenness centrality) as well as its integration and segregation properties (clustering coefficient and local efficiency respectively). Local strength and betweenness centrality are two indices of centrality that measure the relative importance of a node within a network (Zuo et al., 2012). Nodes with high levels of centrality are thought to facilitate information routing in the network with a key role in the overall communication efficiency of a network. The node’s strength is the simplest measure of centrality and is defined as the sum of all the edge weights between a node and all the other nodes in the network. Regions with high nodal strength have high connectivity with other nodes. Betweenness centrality of a node is defined as the fraction of all shortest paths in the network that contain a given node. If a node displays high betweenness centrality it participates in a large number of shortest paths and have an important role in the information transfer within a network. Along with centrality measures, the nodes of a network may display different levels of segregation and integration of information (Sporns, 2013). For example, the clustering coefficient is a commonly used metric to assess the segregation properties of a network. It reflects the ability of a node to communicate with other nodes with which it shares direct connections; in other words, it represents the fraction of triangles around an individual node. It is equivalent to the fraction of the node’s neighbours that are also neighbours of each other (Watts & Strogatz, 1998) and in the case of weighted networks it is calculated as the geometric mean of all triangles associated with each node (Onnela, Saramäki, Kertész, & Kaski, 2005). Finally, an efficient information transfer across distributed nodes (i.e., nodes that are not directly connected) can be quantified via the local path length and local efficiency. In the case of a weighted network, high levels of correlations between the functional activity of two nodes are interpreted as short local path length. The local efficiency is the average of the inverse local path length. Local efficiency is calculated as the global efficiency of the subgraph formed by the node’s neighbours (Boccaletti et al., 2006). It measures the ability of parallel information transfer at local level.

### Global network analyses

Global graph metrics describe the topology of a network with a single number that represents the overall organization of a network. As global measures, we computed the global strength, the global clustering coefficient, and global efficiency (Boccaletti et al., 2006; Rubinov & Sporns, 2010). These measures were calculated as the average of the local strength, local clustering coefficient, and local efficiency of all nodes, respectively.

### Group-level analyses

**Figure 1:**
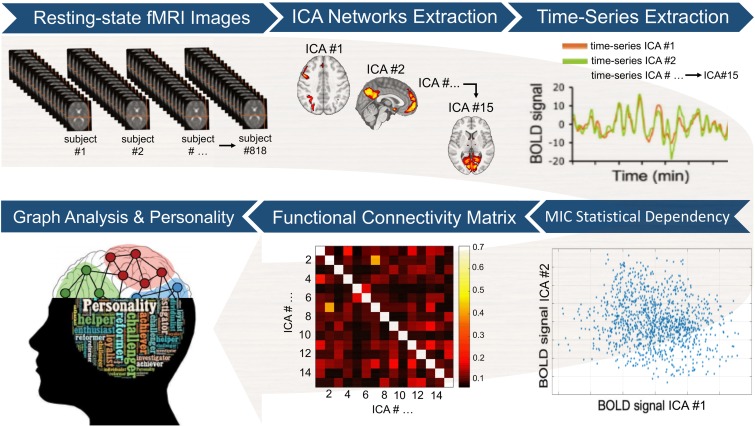
Image analysis workflow. After initial pre-processing, the resting-state functional magnetic imaging (fMRI) data were used to extract a set of 15 separate brain circuits via independent components analysis (ICA). Next, participant-specific time-series from each ICA brain circuit was obtained. The maximal information coefficient (MIC), an index that assesses for linear and nonlinear relationships in big data-sets, was used to measure statistical dependency between each pair of time-series. This led to a 15×15 functional connectivity matrix at the single-participant level. The participant-specific connectivity matrices were then used to compute local and global graph measures (i.e., strength, clustering, efficiency, and betweenness centrality). Each of these graph measures, which quantify different aspects of the brain topological organization, was finally correlated with the five-factor model personality traits at the group level. BOLD=blood-oxygen-level-dependant activity.

To estimate the replicability of our inference framework, the initial sample of *n*=818 participants was randomly split into two sub-samples: a “training” sample (70% of participants, *n*=573) and a “test” sample (30% of participants, *n*=245). The “training” sample was used to examine the association between each of the graph measures (i.e., global and local) and the FFM personality traits. Conversely, the “test” sample was only employed to assess whether the multivariate model based on the “training” sample was able to predict the outcome “connectomic” measures in the “test” sample (i.e., in a group of participants to which the model was completely “agnostic”). To test the associations between graph measures and personality differences, general linear models (GLMs), including each of the FFM traits as well as age and gender as nuisance covariates, were fitted using the “training” sample. The resulting *p* values were corrected for multiple comparisons using a false discovery rate (FDR) procedure. Associations surviving a stringent threshold of *p*<.01 FDR were considered statistically significant. The GLMs fitted in the former procedure were then used to estimate the graph measures resulting in the “test” sample using the demographic and personality scores of the “test” sample as inputs (in other words, the rs-fMRI data of the “train” sample were not employed in this procedure). The similarity between “real” graph measures (i.e., computed using rs-fMRI data from the “test” sample) and “estimated” graph indices (i.e., predicted using the GLMs fitted on “training” data only) was assessed using the relative root mean square error (RRMSE). This approach is typically referred as external validation and tests for generalizability of the findings beyond the study population. The image analysis workflow is summarized in Figure 1.

## Results

### ICA

**Figure 2:**
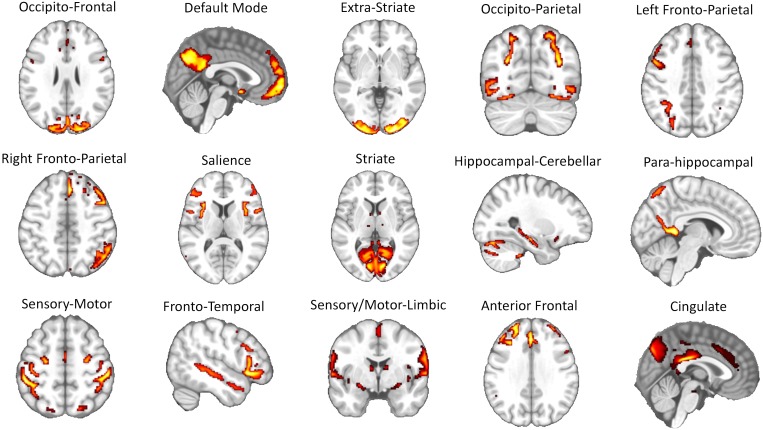
Results of independent component analysis (ICA). A total of 15 separate large-scale functional circuits were identified during the ICA step of the image analysis pipeline (see Figure 1 and methods section in the main text for further details). Each of these circuits was successively used as “node” in the graph analysis. The list of the brain areas belonging to each network is reported in Supplementary Table 1.

The 15 brain networks identified via ICA were represented by a series of circuits that have been consistently reported in past rs-fMRI studies (e.g., the sensory-motor circuit, visual circuits, DMN, left and right fronto-parietal circuits, salience network, etc.) (Raichle, 2015; Toschi, Duggento, & Passamonti, 2017) (see Figure 2 and Supplementary Table 1 for the list of the anatomical regions involved in each network node).

### Correlations between *global* graph indices and FFM traits

No significant associations were found between any of the FFM personality traits and: (i) the global strength (*R*’s<.084, *p*’s>.14); (ii) global clustering coefficient (*R*’s<.081, *p*’s>.15,); and (iii) global efficiency (*R*’s<.083, *p*’s>.17).

### Correlations between *local* graph indices and FFM traits

#### Neuroticism

No associations, either positive and negative, were found between neuroticism scores and: (i) the nodal strength (*R*’s<.07, *p*’s>.75); (ii) local clustering coefficient (*R*’s<.06, *p*’s>.88); (iii) local efficiency (*R*’s<.07, *p*’s>.82); and (iv) betweenness centrality (*R*’s<.09, *p*’s>.59)

### Extraversion

As for neuroticism, no statistically significant association was found between extraversion scores and: (i) the nodal strength (*R*’s<.11, *p*’s>.09); (ii) local clustering coefficient (*R*’s<.12, *p*’s>.04); (iii) local efficiency (*R*’s<.12, *p*’s>.09); and (iv) betweenness centrality (*R*’s<.11, *p*’s>.09).

### Openness

No positive or negative associations were detected between openness scores and: (i) the nodal strength (*R*’s<.07, *p*’s>.97); (ii) local clustering coefficient (*R*’s<.06, *p*’s>.96); (iii) local efficiency (*R*’s<.06, *p*’s>.99); and (iv) betweenness centrality (*R*’s<.09, *p*’s>.27).

### Agreeableness

No positive or negative associations were detected between agreeableness scores and: (i) the nodal strength (*R*’s<.10, *p*’s>.13); (ii) local clustering coefficient (*R*’s<.10, *p*’s>.12); (iii) local efficiency (*R*’s<.10, *p*’s>.15); and (iv) betweenness centrality (*R*’s<.08, *p*’s>.25).

### Conscientiousness

**Figure 3:**
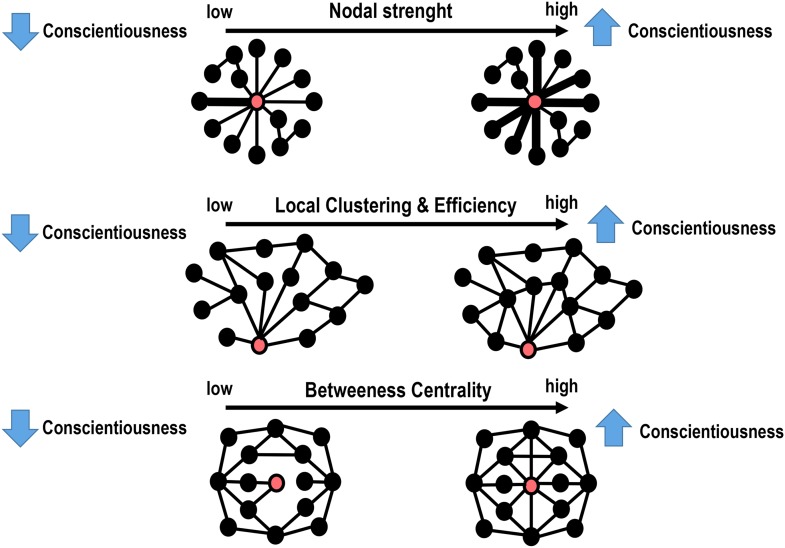
Schematic representation of the main results. Depending on the graph metric (Table 2), the red circle represents either the left or right fronto-parietal network (FPN) or the default mode network (DMN), whereas the black circles represents the 14 remaining network nodes. Top row: The thicker lines in individuals with high levels of conscientiousness indicate the existence of higher strength in the “communications” between the left FPN and the other brain networks. Middle row: People scoring higher in conscientiousness show a higher degree of inter-connectedness between the left FPN and DMN and the local networks consisting of direct neighbours of the left FPN and DMN. Bottom row: The DMN and right FPN have higher betweenness centrality in individuals with higher levels of conscientiousness. This means that the DMN and right FPN are “hub” nodes in conscientious people.

A schematic representation of the significant associations between conscientiousness scores and the local graph measures is illustrated in Figure 3, whereas the statistical details are reported in Table 2. In summary, significantly *positive* correlations were found between conscientiousness scores and the local strength, local clustering coefficient, and local efficiency in the left fronto-parietal network (FPN) (*R*’s>.14, *p*’s<.01, FDR). Increased local clustering and betweenness centrality in the DMN and right FPN were also associated with higher levels of conscientiousness (*R*’s>.14, *p*’s<.005, FDR). External validation showed good replicability, with RRMSE values of around .15 in the “test” sample.

**Table 2:** Positive correlations between *local* graph metrics and conscientiousness scores

Local graph metric	Circuit	Mean (±SD)	*T* score	Pearson’s *R*	*p* (FDR)	RRMSE
Nodal strength	Left FPN	2.37±0.11	3.46	.14	.009	.16
Local clustering	DMN	0.15±0.008	3.40	.14	.008	.15
	Left FPN	0.13±0.007	3.26	.14	.008	.14
Local efficiency	Left FPN	0.09±0.007	3.53	.15	.006	.15
Betweenness centrality	DMN	2.17±2.44	3.68	.15	.002	.16
	Right FPN	0.23±0.79	3.66	.15	.002	.16

*Note*: FDR=false discovery rate; RRMSE=relative root mean square error; FPN=fronto-parietal network; DMN=default mode network.

To further explore which specific aspects of conscientiousness were linked to local graph measures, we conducted post hoc analyses that included conscientiousness facets (i.e., Order, Dutifulness, Achievement striving, Self-Discipline) as main outcome measures. As in the previous analyses, age, sex, and the other FFM traits were included in the GLM as nuisance covariates. We found that betweenness centrality in the DMN was positively associated with Dutifulness (*p*=.01, FDR, RRMSE=.17) and Achievement (*p*=.01, FDR, RRMSE=.16). Finally, betweenness centrality in the right FPN was positively associated with Dutifulness (*p*=.01, FDR, RRMSE=.16).

## Discussion

This study provides compelling new evidence that local graph metrics based on resting-state functional imaging are significantly associated with conscientiousness in a group of 818 young adults drawn from the HCP. More specifically, we found higher nodal strength, local clustering, and local efficiency in the left FPN in people scoring higher in conscientiousness. Likewise, higher local clustering and betweenness centrality in the right FPN and DMN were positively related to conscientiousness scores. A validation approach based on a “training” and “test” split of the total data set supported the robustness, replicability, and “cross-validity” of these findings.

Overall, our results demonstrated the value of applying connectomic approaches to study large-scale functional connectivity patterns in relation to the FFM of personality. The multivariate analyses also showed that the positive association between the FPN/DMN connectivity patterns and conscientiousness was not dependent on other FFM personality traits (i.e., neuroticism, extraversion, openness, and agreeableness) or potentially confounding factors like gender and age variability. Similarly, the non-significant correlations with global connectomic measures (e.g., global clustering and efficiency) suggests that individual differences in conscientiousness are mediated by specific functional dynamics across distinct large-scale neural nodes. In the following sections, we discuss the implication of our findings to improve the understanding of the brain underpinnings of conscientiousness as well as the main strengths and limitations of the study.

### FPN and DMN connectivity patterns mediate conscientiousness

The higher nodal strength in the left FPN in people scoring high in conscientiousness reflects the fact that this specific circuit “node” has heightened “communications” with the other nodes. Highly conscientious people also show higher local clustering in the left FPN, which implies that the FPN is densely interconnected to its neighbours and formed an elevated number of local aggregates (“triangles”) with its most adjacent nodes. At the same time, the local efficiency in the left FPN and the betweenness centrality in the right FPN were higher in people scoring higher in conscientiousness.

The FPN includes citoarchitecturally complex and evolutionarily recent cortices that have been associated with inter-participants variance in several cognitive measures (Mueller et al., 2013; Zilles, Armstrong, Schleicher, & Kretschmann, 1988). Furthermore, a study in *n*=126 people from the HCP database reported that the functional connectivity patterns involving the FPNs were the most distinguishing features (“fingerprints”) that predicted variability in cognitive functioning across individuals (Finn et al., 2015). Although the FPNs are typically engaged during tasks that require high levels of attention and cognitive control, their connectivity patterns *at rest* also predict participant-specific cognitive performance with a high degree of precision (Finn et al., 2015; Miranda-Dominguez et al., 2014). This may depend on the fact the FPN nodes act as flexible “hubs” to coordinate the activity of several other brain networks (Finn et al., 2015; Miranda-Dominguez et al., 2014).

The enhanced connectivity patterns of FPNs in people scoring high in conscientiousness can therefore be interpreted as a “sign” of increased cognitive control in these individuals, bearing in mind the shortcomings of making reverse inferences (Poldrack, 2006). This is in keeping with several observations showing that conscientious people are efficient in pursuing their objectives, which is itself a critical predictor of academic or occupational success, healthy life-styles, and longevity (Noftle & Robins, 2007; Ozer & Benet-Martinez, 2006; Roberts, Lejuez, Krueger, Richards, & Hill, 2014; Sutin et al., 2016). Our data are also consistent with past neuroimaging studies that have implicated the dorsolateral prefrontal cortex (DLPFC) and other prefrontal cortex areas (e.g., the anterior cingulate cortex (ACC), which is also part of the FPN) in conscientiousness (Bunge & Zelazo, 2016; DeYoung et al., 2010; Forbes et al., 2014; Jackson, Balota, & Head, 2011; Kapogiannis et al., 2012; Matsuo et al., 2009; Whittle et al., 2009). Nevertheless, our results show that it is the FPN *connectivity patterns* with the other “nodes” which is linked to conscientiousness rather than the activity in the DLPFC/ACC in isolation. This is a key issue, especially when considering the necessity to progress from models of personality that consider the function of single brain regions, to more naturalistic frameworks that describe individual differences in behavioural traits in terms of large-scale networks’ dynamics.

Finally, we found that the DMN showed higher local clustering and betweenness centrality in relation to high conscientiousness scores. This finding was predicted on the basis of previous data showing that connectivity patterns involving the DMN predict variability in a single “positive-to-negative” behavioural axis (Smith et al., 2015). The DMN also contributes to working memory performances via the dynamic reconfiguration of its interactions with other networks, which suggests that the DMN is actively involved during the execution of cognitively demanding tasks (Vatansever, Menon, Manktelow, Sahakian, & Stamatakis, 2015). Overall, high-level cognitive functioning is critical in human evolution and is central in the life of conscientious people. Hence, we speculate that enhanced DMN “interplay” with other nodes explains, in mechanistic terms, why conscientious individuals are able to efficiently elaborate complex plans like imaging and planning future scenarios. This hypothesis is supported by our post hoc analyses showing that local measures in the DMN (i.e., local clustering and betweenness centrality) are respectively linked to the Dutifulness facet (i.e., reliable, dependable, careful, scrupulous, and strictly adherent to rules) and Achievement Striving facet (i.e., industrious, enterprising, ambitious, purposeful, and driven) of conscientiousness.

### Strengths and limitations

The main strengths of our study are: (i) the large, homogeneous, and well-characterized sample of participants in terms of FFM personality traits, demographic variables, and neuroimaging data, which *in itself* offers greater statistical power compared with several previous studies, and (ii) the fact that we employed robust statistical approaches to show specificity and replicability of our findings. We note, however, that the effects sizes were small (*T*′s~3.5), although in the typical range of other studies using similar sample sizes (Mackey et al., 2016; Smith et al., 2015). There was also a relatively high number of statistical tests, although we strived to attenuate this potential problem with the use of stringent statistical procedures to correct for multiple comparisons (*p*<.01, FDR).

The fact that conscientiousness was the sole personality trait related to “connectomic” metrics *does not* necessarily imply that the other FFM traits *do not* have such brain correlates. Several reasons why the other FFM traits were not related to functional connectomic indices may be speculated—even if not resolved by our data set. These include: (i) type II errors; (ii) non-linear relationships between personality traits and brain connectomic metrics; (iii) the fact that our group-level statistical models were multivariate rather than univariate, which means that the shared variance explained by the other FFM traits was factored out while analysing the effect of each FFM trait; (iv) the possibility that correlations between brain functional connectomic measures and other personality traits *do exist* but can only be revealed by “meta-trait” measures (DeYoung, Peterson, & Higgins, 2002).

Perhaps more importantly, our study suggests that different neuroimaging modalities and analytical techniques may be able to reveal the unique nature of *how* the brain mediates each of the FFM traits. Consistent with this idea, we have recently found in *n*=507 individuals from the same HCP data set that measures of cortical anatomy (i.e., cortical thickness, folding, and surface area) were differently associated with each of the FFM traits (Riccelli, Toschi, Nigro, Terracciano, & Passamonti, 2017). Hence, brain structural heterogeneity is likely to underlie variability in all FFM traits, whereas the same may not be true for functional measures that assess more transient “communication” patterns. Different functional connectivity approaches (e.g., time-variant connectivity methods) are also warranted to further explore the complexity of the neural dynamics mediating individual differences in personality (Riccelli, Passamonti, Duggento, Guerrisi, Indovina, Terracciano, et al., 2017a; Riccelli, Passamonti, Duggento, Guerrisi, Indovina, & Toschi, 2017b).

### Summary and conclusions

To summarize, we found robust and specific associations between conscientiousness and graph measures of local connectivity in the FPN and DMN. These highly integrated circuits include different parts of the prefrontal and parietal cortices, a set of brain regions that have significantly evolved in human beings and have been consistently implicated in goal-setting and planning, two high-order cognitive functions in which conscientious people excel.

## Supplementary material

For supplementary material accompanying this paper visit http://dx.doi.org/10.1017/pen.2017.2. click here to view supplementary material
