# Supplementary material for: Functional Connectome of the Five-Factor Model of Personality
Source: Personal Neurosci. 2018 May 25;1:e2. doi: 10.1017/pen.2017.2 (PMC6171528; doi:10.1017/pen.2017.2)
Supplement: Supplementary file 1 [file S2513988617000025sup001.pdf]

Supplementary Table 1. Anatomical Localization of topologically connected clusters in each of the 15 Independent Component Analysis (ICA) components after thresholding at the 99th percentile. MAX: maximum value of dual regression coefficient within specific cluster. MNI: Montreal Neurological Institute. All coordinates are in mm. L, left hemisphere, R, right hemisphere.

**ICA #1**<br>**Occipito-Frontal Circuit**	N° of voxels	MAX	MNI X	MNI Y	MNI Z
Visual cortex V2 BA18 R	1326	62.2	14	-92	30
Caudate R	291	63.7	10	0	12
Visual cortex V4 R	239	42.4	24	-74	-16
Visual cortex V3V L	150	39.9	-20	-70	-14
Amygdala superficial group L	147	56.2	-24	0	-10
Caudate L	145	62.4	-8	2	8
Amygdala superficial group R	67	57.3	24	2	-10
Para-Cingulate Gyrus	55	29.6	0	32	32
Inferior Frontal Gyrus BA44 R	39	30.7	52	10	26
Brain stem	36	40.4	6	-10	-16
Inferior Frontal Gyrus BA45 L	34	34.9	-48	16	-6
Cerebellum L	33	42.5	-2	-52	-34
Frontal operculum R	21	35.5	50	18	-6
Putamen R	20	28	22	6	6
Frontal Operculum L	15	33	-32	20	10
Premotor cortex BA6 R	15	28.3	2	18	50
Lateral geniculate body L	14	35.1	-22	-26	-8
Inferior Frontal Gyrus BA44 L	13	28.8	-50	6	24
Cerebellum R	13	29.8	18	-42	-46
Lateral Occipital Cortex L	12	28.4	-48	-82	4

**ICA #2**<br>**Default Mode Network**	N° of voxels	MAX	MNI X	MNI Y	MNI Z
Frontal Pole / medial prefrontal cortex	1285	73.4	0	62	-6
Posterior Cingulate Gyrus L	865	70.4	-2	-52	16
Inferior parietal lobule L	472	73.5	-46	-68	44
Frontal Pole R	48	49.7	18	42	50
Inferior parietal lobule R	47	46.2	54	-64	36
Subgenual Anterior Cingulate Cortex	26	74.4	0	6	-12
Inferior frontal gyrus BA45 L	18	44.7	-40	30	-14

**ICA #3**<br>**Extra-striate Visual Circuit**	N° of voxels	MAX	MNI X	MNI Y	MNI Z
Occipital Gyrus L	1424	65.7	-26	-86	-20
Occipital Gyrus R	1382	65	26	-94	0

**ICA #4**<br>**Occipito-Parietal Circuit**	N° of voxels	MAX	MNI X	MNI Y	MNI Z
Inferior parietal lobule R	1073	57.8	34	-86	28
Visual cortex V2 BA18 L	772	57.1	-26	-88	26
Visual cortex V2 BA18 R	488	45	30	-56	-6
Visual cortex V4 L	457	39.9	-28	-64	-6
Premotor cortex R	11	27.6	52	8	38

ICA #5<br>Left Fronto-Parietal Circuit	N° of voxels	MAX	MNI X	MNI Y	MNI Z
Dorsolateral prefrontal cortex L	1619	79.7	-52	14	32
Inferior parietal lobule L	605	48.2	-40	-56	56
Medial Superior Frontal Gyrus L	172	55.5	-2	26	46
Inferior Frontal Gyrus BA45 R	89	35.5	48	32	20
Superior Frontal Gyrus L	88	47.6	-28	14	62
Frontal Orbital Cortex L	46	46	-34	36	-10
Medial Frontal Pole R	32	36.5	2	58	14
Inferior Temporal Gyrus L	30	34.2	-54	-44	-12
Middle Temporal Gyrus L	23	29.5	-62	-54	-8
Inferior Frontal gyrus BA44 L	18	30.1	-38	22	-2
Cerebellum R	16	33	10	-78	-30
Inferior Temporal Gyrus L	16	29.3	-50	-60	-20

ICA #6<br>Right Fronto-Parietal Circuit	N° of voxels	MAX	MNI X	MNI Y	MNI Z
Frontal Pole R	1939	63	42	56	0
Inferior parietal lobule R	701	47.7	42	-62	52
Cerebellum L	30	32.6	-8	-78	-30
Inferior Frontal Gyrus BA45 R	30	30.1	56	22	4
Cerebellum L	24	29.2	-36	-68	-40
Middle Temporal Gyrus R	23	30.8	64	-28	-2
Frontal Orbital Cortex L	12	30.9	-32	36	-10

ICA #7<br>"Salience" Network	N° of voxels	MAX	MNI X	MNI Y	MNI Z
Supra-marginal Gyrus L	535	50	-60	-36	32
Insula L	474	67.1	-40	-12	-8
Inferior parietal lobule R	434	60.1	56	-30	34
Inferior Frontal Gyrus BA44 R	418	74.2	58	12	0
Inferior Frontal Gyrus BA45 L	212	41	-48	42	12
Pre-motor cortex BA6 R	191	38.8	2	12	42
Frontal Pole R	129	43.6	50	44	4
Superior parietal lobule R	101	48.1	16	-34	42
Insula R	100	68.5	42	-8	-10
Superior parietal lobule L	81	51.6	-14	-30	38
Orbitofrontal cortex L	35	36.3	-32	38	-10
Middle Temporal Gyrus L	33	32.3	-60	-62	0
Anterior Cingulate Cortex L	18	30.8	-10	8	38
Pre-motor cortex BA6 L	17	28.4	-2	6	66
Amygdala L	<15	26.8	-20	0	-14
Amygdala R	<15	25.9	22	2	-14

ICA #8<br>Striate Visual Circuit	N° of voxels	MAX	MNI X	MNI Y	MNI Z
Intra-Calcarine Cortex R / L	2716	134	10	-72	14
Thalamus L	20	60.8	-12	-32	-4
Thalamus L	19	58	-10	-16	8
Thalamus R	17	60.4	14	-30	-4

ICA #9<br>Hippocampal-Cerebellar Circuit	N° of voxels	MAX	MNI X	MNI Y	MNI Z
Cerebellum L	1439	41.4	-34	-68	-22
Thalamus L	267	49.7	-10	-4	14
Hippocampus R	249	31.9	26	-20	-16
Hippocampus subiculum L	221	34.8	-14	-38	2
Insula L	115	30.3	-40	-20	-2
Secondary somatosensory cortex / Parietal operculum R	111	32.2	36	-28	20
Thalamus	42	34.8	0	-24	-2
Insula L	33	24.8	-30	22	-6
Cerebellum R	32	35.7	30	-40	-40
Insula R	30	23	32	22	-4
Cerebellum L	25	28.1	-32	-42	-40
Caudate L	22	22.3	-16	6	22
Caudate R	19	25.3	18	4	24
Hippocampus subiculum R	16	23.2	12	-30	-2
Cerebellum R	14	28.6	16	-44	-46
Brain-Stem	13	26.1	0	-34	-8
Cerebellum L	12	29.6	-12	-46	-44
Frontal Operculum R	11	21.3	50	18	-6

ICA #10<br>Para-hippocampal Circuit	N° of voxels	MAX	MNI X	MNI Y	MNI Z
Posterior Cingulate Gyrus R	608	72.8	8	-48	4
Superior parietal lobule L	510	42.4	-6	-66	62
Precuneus L	403	69.1	-16	-58	18
Middle Frontal Gyrus R	319	43.2	28	32	52
Inferior parietal lobule L	301	55.2	-34	-84	38
Inferior parietal lobule R	253	54	42	-80	32
Premotor cortex BA6 L	127	35.6	-22	10	58
Parahippocampal Gyrus L	113	54	-32	-40	-12
Parahippocampal Gyrus R	92	47.5	34	-36	-14
Hippocampus subiculum R	17	37.2	22	-20	-16
Cerebellum L	16	42.5	-10	-48	-44
Posterior Cingulate Cortex	13	32.7	0	-24	28
Cerebellum R	12	35.3	12	-46	-44

ICA #11<br>Sensory-Motor Circuit	N° of voxels	MAX	MNI X	MNI Y	MNI Z
Primary somatosensory cortex BA2 L	1353	46.3	-52	-30	52
Primary somatosensory cortex BA2 R	769	46.3	56	-24	52
Premotor cortex BA6 R	343	36.4	40	-22	66
Premotor cortex BA6 L	91	32.3	-54	8	38
Inferior Frontal Gyrus BA44 R	37	25.6	54	10	24
Secondary somatosensory cortex / Parietal operculum L	36	24.7	-50	-20	20
Medial Premotor cortex BA6	28	22.2	0	-6	56
Secondary somatosensory cortex / Parietal operculum L	27	38.5	-38	-4	16
Superior parietal lobule R	24	22.3	18	-70	54
Secondary somatosensory cortex / Parietal operculum R	20	33.3	40	-2	16
Secondary somatosensory cortex / Parietal operculum R	15	21	52	-16	18

Superior parietal lobule L	14	20.5	-16	-72	54

**ICA #12 Fronto-Temporal Circuit**	N° of voxels	MAX	MNI X	MNI Y	MNI Z
Broca's area BA45 L	868	72.9	-48	20	-6
Middle Temporal Gyrus L	526	46	-48	-32	-2
Premotor cortex BA6 L	468	44	-4	24	64
Middle Temporal Gyrus R	392	50.1	48	-22	-8
Broca's area BA45 R	309	46.3	50	22	-6
Temporal Pole L	85	32.9	-48	2	-24
Premotor cortex BA6 L	52	33.1	-44	2	56
Superior Frontal Gyrus L	48	32.1	-2	54	30
Broca's area BA45 R	33	31	54	22	28
Inferior parietal lobule L	15	29.5	-42	-54	22

**ICA #13 Sensory/Motor-Limbic Circuit**	N° of voxels	MAX	MNI X	MNI Y	MNI Z
Primary Somatosensory Cortex (BA3)	1232	125	52	-6	26
Primary Somatosensory Cortex (BA3)	1220	119	-54	-8	24
Pre-motor Cortex (BA6)	138	39.1	2	2	64
Secondary Somatosensory Cortex /Parietal Operculum (OP2)	81	55.6	-34	-30	18
Amygdala, superficial group	30	57.9	26	0	-10
Amygdala, superficial group	26	50.7	-26	-2	-10
Caudate Nucleus	25	51.5	10	0	12
Caudate Nucleus	24	41.7	-8	2	8
Primary Motor Cortex (BA4)	19	32.5	20	-28	58
Primary Motor Cortex (BA4)	15	41	-18	-30	60

**ICA #14 Anterior Frontal Circuit**	N° of voxels	MAX	MNI X	MNI Y	MNI Z
Middle Frontal Gyrus L	1608	41	-36	34	40
Paracingulate Gyrus L	425	39.2	-2	28	42
Frontal Pole R	196	30.4	32	52	28
Inferior parietal lobule L	179	29.2	-54	-50	48
Premotor cortex BA6 R	85	27.7	18	12	68
Inferior Frontal Gyrus BA45 R	75	24.4	54	24	28
Frontal Pole R	49	24.5	2	60	-6
Inferior Frontal Gyrus BA45 L	35	30.9	-50	16	-4
Amygdala superficial group R	25	31	16	-4	-14
Medial Precentral Gyrus	21	24.8	0	-34	48
Amygdala superficial group L	14	26.8	-16	-2	-16
Frontal Pole L	14	26.4	-30	50	-12
Subgenual Anterior Cingulate Cortex	11	28.7	0	6	-12

**ICA #15 Cingulate Cortex Circuit**	N° of voxels	MAX	MNI X	MNI Y	MNI Z
Posterior Cingulate Gyrus	2353	114	0	-30	26
Paracingulate Gyrus R	138	32.4	2	24	36
Posterior Cingulate Gyrus L	110	38.5	-14	-46	34

Frontal Operculum R	56	37.8	58	12	-2
Anterior intra-parietal sulcus L	34	27.6	-34	-58	40
Frontal Operculum L	26	32.1	-58	8	0
Medial Precentral Gyrus	21	30.8	0	-34	48
Insula R	12	28.8	42	-8	-10
Paracingulate Gyrus L	12	27.1	-10	26	28
